# Dependence of Fluorescence Quenching of CY3 Oligonucleotide Conjugates on the Oxidation Potential of the Stacking Base Pair

**DOI:** 10.3390/molecules25225369

**Published:** 2020-11-17

**Authors:** Jens Sobek, Ralph Schlapbach

**Affiliations:** Functional Genomics Center Zurich, Eidgenössische Technische Hochschule (ETH) Zurich and University of Zurich, 8057 Zurich, Switzerland; ralph.schlapbach@fgcz.ethz.ch

**Keywords:** CY3, fluorescence quenching, short oligonucleotides, hybrid stabilisation, oligonucleotide dye conjugates, dye effect, single-molecule fluorescence, hybridisation, guanine oxidation

## Abstract

To understand the complex fluorescence properties of astraphloxin (CY3)-labelled oligonucleotides, it is necessary to take into account the redox properties of the nucleobases. In oligonucleotide hybrids, we observed a dependence of the fluorescence intensity on the oxidation potential of the neighbouring base pair. For the series I < A < G < 8-oxoG, the extent of fluorescence quenching follows the trend of decreasing oxidation potentials. In a series of 7 nt hybrids, stacking interactions of CY3 with perfect match and mismatch base pairs were found to stabilise the hybrid by 7–8 kJ/mol. The fluorescence measurements can be explained by complex formation resulting in fluorescence quenching that prevails over the steric effect of a reduced excited state trans-cis isomerisation, which was expected to increase the fluorescence efficiency of the dye when stacking to a base pair. This can be explained by the fact that, in a double strand, base pairing and stacking cause a dramatic change in the oxidation potential of the nucleobases. In single-molecule fluorescence measurements, the oxidation of G to 8-oxoG was observed as a result of photoinduced electron transfer and subsequent chemical reactions. Our results demonstrate that covalently linked CY3 is a potent oxidant towards dsDNA. Sulfonated derivatives should be used instead.

## 1. Introduction

**Figure 1:**
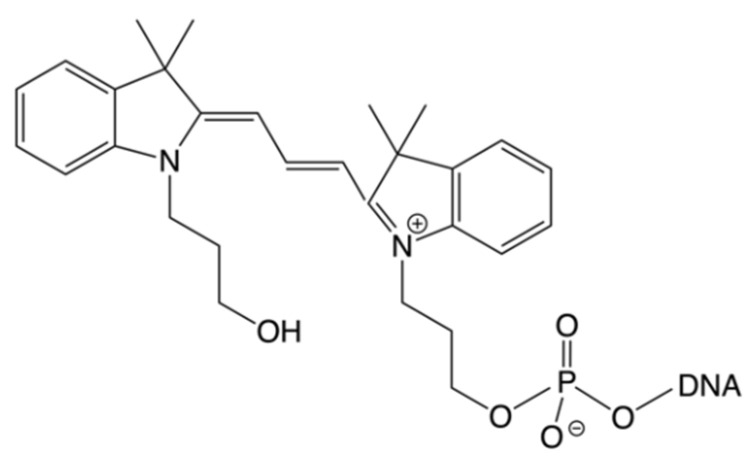
Molecular structure of astraphloxin (CY3) oligonucleotide conjugates.

Astraphloxin, better known under the name of CY3 (Figure 1), and its sulfonated derivatives linked to nucleosides and oligonucleotides are used in a large variety of analytical applications. The photophysical properties of the dyes are well investigated by static and time-resolved methods, including single-molecule applications [1,2,3,4,5,6,7,8,9,10,11,12,13,14,15,16,17,18,19,20]. The main deactivation pathway of the first excited state of CY3 proceeds via a radiationless transition, the trans-cis isomerisation of an exocyclic double bond, which decreases the fluorescence quantum yield to less than 10% [3,5,7,9,21,22,23,24]. Blocking the isomerisation by conjugation with nucleosides, oligonucleotides, and proteins, the chemical modification of the chromophore as in CY3B, or the use of a highly viscous solvent, increases the quantum yield up to 85% [3,9,25,26,27]. Upon hybridisation of CY3 oligonucleotide conjugates and allowing the hydrophobic dye to stack to a nucleobase pair, the fluorescence efficiency was found to be reduced, an effect for which no satisfactory explanation was given to date. In nucleobases and oligonucleotides conjugated to various dyes and aromatic hydrocarbons, quenching by photoinduced electron transfer (PET) was found to play a crucial role in their fluorescence characteristics [28,29,30,31,32].

The interpretation of fluorescence data of CY3 conjugated to DNA is complicated by the existence of a dynamic equilibrium between non-stacking and a larger number of stacking conformations, which decrease the isomerisation efficiency by blocking molecular movements [3,33]. The analysis of the fluorescence characteristics was hampered by the fact that partly unstacked conformations were found that allow isomerisation [34]. This manifests through a complex fluorescence decay that consists of three components, and reflects the interactions of the dye with the nucleobase pair [3,8,9]. Stacking interactions with dangling nucleotides, dyes and other aromatic systems were found to increase the stability of short oligonucleotide hybrids similar to the effect of an additional base pair [19,35,36,37].

In this article, we focus our attention on the observation that fluorescence of CY3-labelled oligonucleotides is quenched upon hybridisation depending on the nature of the neighbouring base pair. It is a known fact that the close proximity of an electron donor and an acceptor can result in fluorescence quenching by electron transfer, and that many dyes are good electron acceptors [29,38,39,40]. However, this obvious explanation has not been taken into consideration to explain fluorescence properties of CY3 oligonucleotide conjugates. The reason for this is that the free energy of electron transfer, ΔG_ET_, was found to have no negative free energy using the oxidation potential of guanine, the nucleobase with the lowest oxidation potential [29], and an increase in fluorescence in Stern-Volmer quenching experiments [39]. However, experimental and theoretical studies have shown that the oxidation potential of guanine is strongly decreased by base pair formation and stacking interactions as a result of intra-base proton transfer and delocalisation of the charge [41,42,43]. Voltammetric measurements in dry aprotic solvents by the group of Peluso have shown a decrease in the oxidation potential for the guanine cytosine base pair by 0.34 eV, in good agreement with values of 0.28 eV and 0.3 eV, respectively, calculated by Crespo-Hernadez and Paukku [44,45,46,47]. Calculations by Hutter and Colson have demonstrated a strong decrease in ionisation energies upon base paring by 0.58 to 0.75 eV, respectively, due to the formation of the G neutral radical by proton transfer to C [43,48,49]. For the AT base pair, a corresponding stabilisation by proton transfer was not found. An experimental verification by the determination of redox potentials of oligonucleotides and hybrids in aqueous solution is complicated by fast secondary reactions upon oxidation and reduction, including intramolecular electron transfer, intra-base pair proton transfer, and reaction with water, resulting in irreversible cyclic voltammograms [29,50]. This explains the lack of fundamental electrochemical data that prevents a precise determination of free energies of electron transfer, for which experimental or calculated ionization energies were used instead.

In a previous publication, we have shown that the secondary reactions compromising cyclic voltammograms can be investigated by single-molecule fluorescence measurements. For hybridisation of short dye-labelled oligonucleotides to probes immobilised in zero-mode waveguide nanostructures, we observed, among other effects, the transfer of the positive charge from G^+^ to a distant G dimer, and the formation of 8-oxoG and a variety of other reaction products in agreement with findings of other groups [51,52,53,54,55,56,57].

Here, we demonstrate that a correlation exists between CY3 fluorescence efficiency and the donor properties of the stacking nucleobase pair in ds DNA. Our data suggest that the reduction in fluorescence efficiency upon hybridisation is caused by donor–acceptor interactions due to the formation of a weak ground state complex, and fluorescence quenching by photoinduced electron transfer [20].

## 2. Results

### 2.1. Optical Spectra

**Figure 2:**
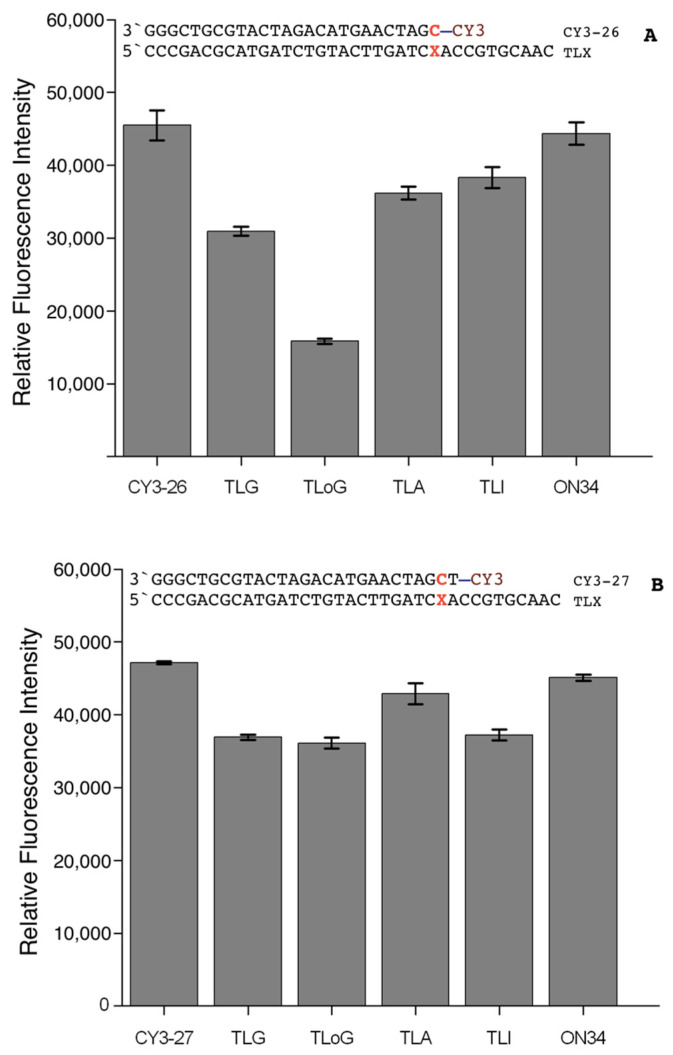
Fluorescence intensity of TLX derivatives in HBS buffer hybridised with (**A**) CY3-26 and (**B**) CY3-27. The dye is stacking to the C:X base pair in CY3-26, and the adjacent T:A base pair in CY3-27.

Absorption and fluorescence spectra of hybrids of CY3-26 and CY3-27, respectively, do not show a significant band shift and broadening compared to the single strand (ss) oligonucleotides (Table 1). Static fluorescence spectra were recorded to investigate the effect of the dye when stacking to structurally similar purine base pairs. G:C and 8-oxoG:C feature the same hydrogen bond pattern, whereas in I the 2-amino group is missing. Nevertheless, nearly 90% of all molecules were found to feature Watson–Crick I:C base pairing [58]. From this, it can be assumed that the structure of the resulting hybrid is similar for C:G, C:8-oxoG, and C:I. Adenine is different, in that it forms a wobble pair with C that is among the least stable mismatches [59,60]. The fluorescence maxima of the CY3-26/TLX hybrids are plotted in Figure 2A, along with the ss controls CY3-26 and CY3-26/ON34. Whereas there is no difference between CY3-26 and non-hybridising CY3-26/ ON34, a clear intensity decrease upon hybridisation between 15% and 65% was observed for the hybrids, following the order TLI > TLA > TLG > TLoG, which parallels the trend of decreasing oxidation potentials [29,61,62]. For comparison, CY3-27/TLX hybrids show only a small reduction in fluorescence intensity upon hybridisation but there is no difference between base pairs except for TLA (Figure 2B).

### 2.2. Surface Plasmon Resonance (SPR) Measurements

**Figure 3:**
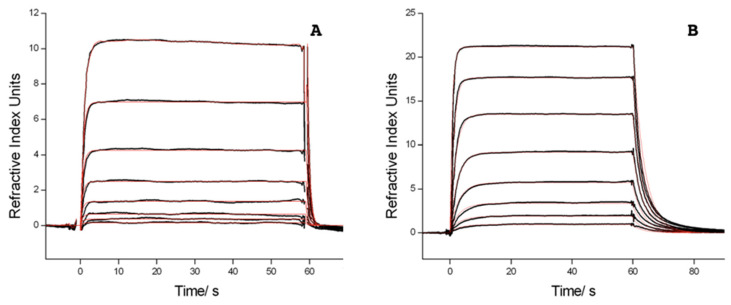
Double referenced sensorgrams (black) for hybridisation of TLoG with (**A**) 7m and (**B**) 3CY3-7m, measured on a CMTEG surface at 20 °C, and fits of a 1 + 1 interaction model (red). Dilution series of 8 concentrations were measured in duplicates, starting at 5 µM and 0.82 µM for 7m and 3CY3-7m, respectively. Only one injection per concentration is shown for clarity.

As a measure of oligonucleotide dye interactions, the increase in affinity upon dye labelling was determined by SPR. Hybridisation of 3CY3-7m to immobilised TL derivatives was measured and compared to the unlabelled compound 7m (Figure 3). Equilibrium intensities were used to calculate *K*_D_. Data in Table 2 show higher affinities for the dye-labelled oligonucleotides that correspond to a relatively small stabilisation by 7–8 kJ/mol, calculated by using the van’t Hoff equation [36]. The stabilisation was slightly lower when CY3 was stacking to a G:G and G:8-oxoG mismatch, respectively.

Furthermore, we investigated the hybridisation of 7nt oligonucleotides labelled with various cyanine and rhodamine dyes, a carbopyronine, fluorescein, and methylene blue, to Bio34G and Bio34r, respectively. For all oligonucleotide dye conjugates, a hybrid stabilisation was observed, except for the highly negatively charged dyes fluorescein, DY-548, and DY-549 (Table 3). A stabilisation was also found for CY3 stacking to a C:U mismatch (5CY3-7n/ Bio34U) and other mismatches (data not shown), and for CY3-7n/ Bio34ap (Table 4). Kinetic data of hybridisation are summarised in Supplement Table S1.

### 2.3. Single-Molecule Measurements

**Figure 4:**
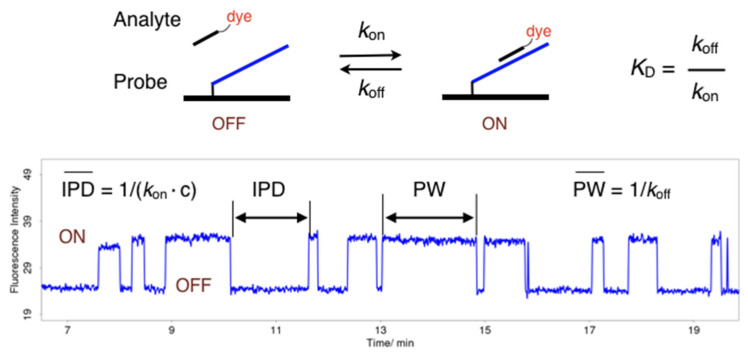
Scheme of the experimental setup.

Single-molecule hybridisation measurements were conducted for more than 40 probe-analyte systems in a zero-mode waveguide (ZMW) nanostructure chip using a modified DNA sequencer (RSII). The working principle and characteristics of the RSII and its modified version, RSII+, was reviewed in the literature [19,63,64,65]. From each measurement, 150,250 fluorescence traces were obtained of which typically 10,000–50,000 were identified by software to arise from a single immobilised molecule in a ZMW, and used for analysis. Due to the large number of traces, pulse pattern and pattern changes could be reliably analysed in detail. The experimental setup is shown in Figure 4.

**Figure 5:**
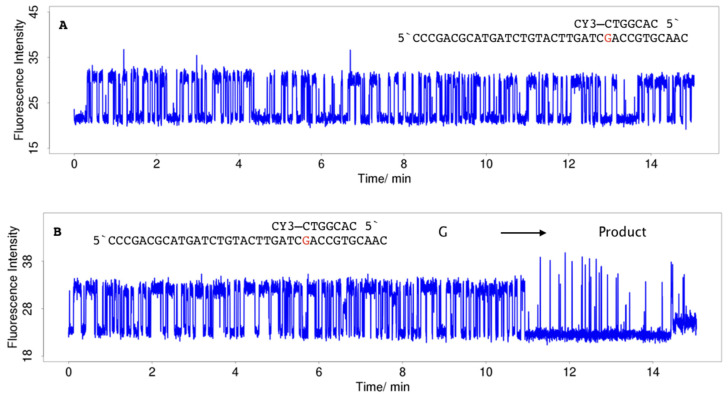

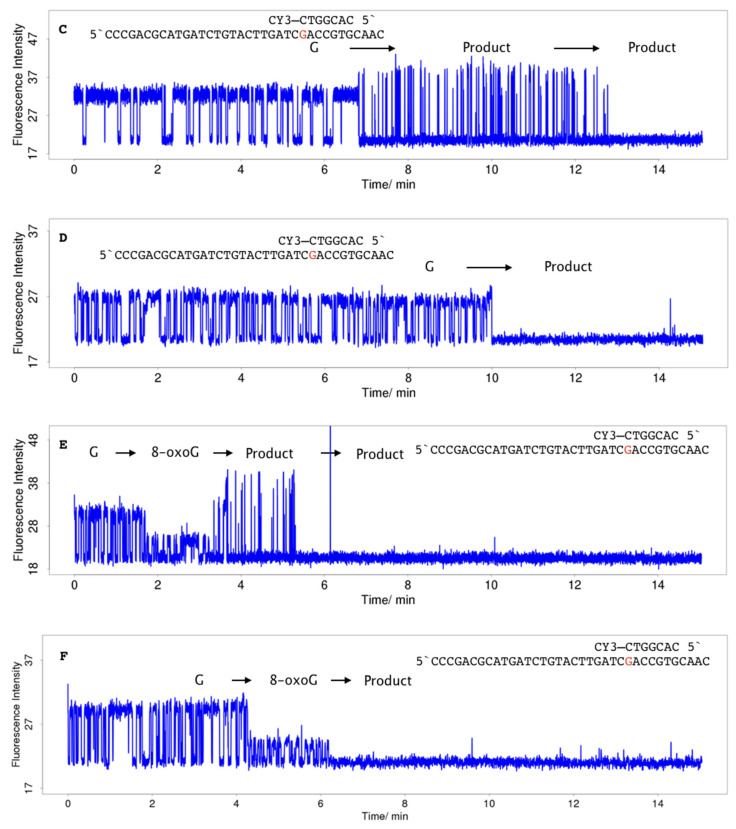
Single-molecule fluorescence traces for hybridisation of 100 nM 3CY3-7m to TLG. (**A**) Continuous train of pulses. (**B**,**C**) Formation of low affinity products due to a reaction within the stacking C:G base pair, revealed by changes of pulse width (PW), inter-pulse duration (IPD), and pulse intensity. (**C**) shows a sequence of two reactions creating another product (termination). (**D**) Termination. Formation of a low affinity product characterised by a loss of affinity. (**E**) Consecutive sequence of reactions. Firstly, formation of 8-oxoG, followed by a secondary reaction creating a low affinity product which is converted to a product having no affinity (termination). (**F**) Formation of 8-oxoG, followed by termination. The reaction sequence indicates that 8-oxoG reacts to a different product compared with the molecule in 4E. The products causing the pulse pattern to terminate are not necessarily identical.

In a single molecule measurement, analyte hybridisation and hybrid dissociation manifest by the appearance of fluorescence pulses which are characterised by the pulse width (PW), the inter-pulse duration (IPD), and fluorescence intensity, that create a pulse pattern characteristic for a probe-analyte pair. Continuous trains of pulses over a longer period of time were observed (Figure 5A). However, in many fluorescence traces (20% to >99%), changes in the initial pulse pattern were found which were attributed to chemical reactions changing the nature of the immobilised probe molecule [20]. The nature and rate of pulse pattern changes were found to depend on the base pair to which the dye was stacking, the sequence context, in particular the presence of guanine within the hybrid and its distance to the stacking dye, and hybridisation conditions including the composition of the hybridisation buffer and additives, and the laser intensity. In short oligonucleotide hybrids, the chemical modification of a single base causes a strong destabilisation as revealed by the transition to shorter pulses (Figure 5B,C) or, in case of a loss of affinity, the termination of the train of pulses (Figure 5C,D). Single-molecule real-time sequencing after hybridisation of CY3-labelled analytes to cyclic 93 nt and 199 nt oligonucleotides, respectively, revealed that in case of termination the molecule was present at the surface (data not shown).

Typically, nucleobase modifications were found to decrease the PW by an order of magnitude, whereas the IPD only slightly changes. This was confirmed by SPR measurements using oligonucleotides having mismatches or modified nucleobases. The only exception was the substitution of G by 8-oxoG in TLG and TLoG, having comparable hybridisation kinetics (Supplement Table S1). In a previous publication, we identified the oxidation of G to 8-oxoG by a comparison of rate constants measured with SPR, and the two-fold decrease in the fluorescence intensity [20]. This is in agreement with results of static fluorescence measurements, demonstrating a similar decrease in CY3 fluorescence upon stacking to a C:8-oxoG base pair (Figure 2A). The characteristic pulse pattern of 8-oxoG was observed in all probe-analyte pairs in which the dye was stacking to a C:G base pair.

Because 8-oxoG is much more reactive than G due to a more than 400 meV lower oxidation potential [66], its pulse pattern changes after seconds or minutes [67]. Secondary reactions lead to the formation of two products, one having a low affinity characterised by short and intense pulses (Figure 5E), and a second product having no affinity that terminates the measurement (Figure 5F). Fluorescence traces can be used to study reaction pathways and the progression of chemical reactions including the formation of short-lived intermediates. Analysis of reaction products is the subject of ongoing work.

**Figure 6:**
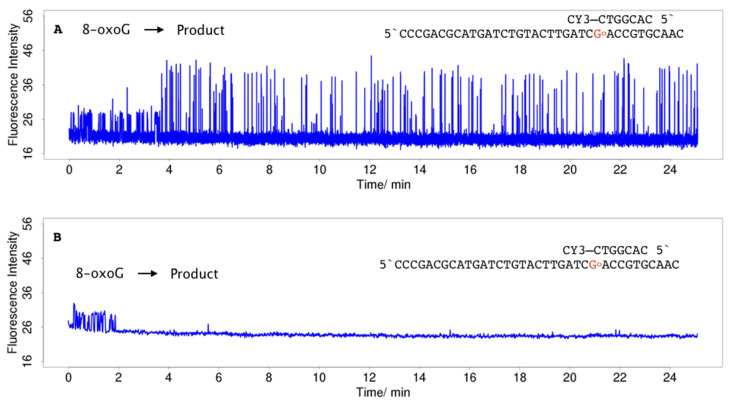
Single-molecule fluorescence traces for hybridisation of 100 nM 3CY3-7m to TLoG. The dye is stacking to a highly reactive C:8-oxoG base pair. (**A**) Formation of a low affinity product characterised by shorter PW, lower IPD, and higher fluorescence intensity. (**B**) In contrast to 4A, the molecule reacts via a different pathway, to a product having no affinity (termination).

**Figure 7:**
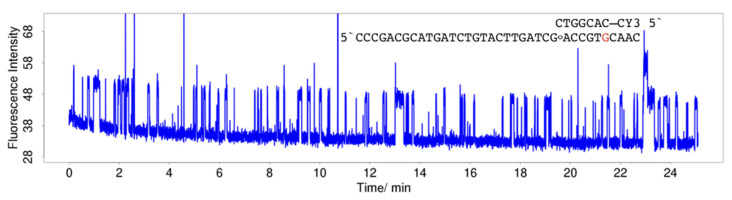
Hybridisation of 20 nM 5CY3-7m to TLoG. Continuous train of pulses. In contrast to hybridisation with 3CY3-7m (Figure 5A,B), the dye is stacking to a C:G base pair which is less prone to photoinduced reactions due to a lower free energy of ET.

The effect of an increase in the driving force can be demonstrated in hybridisation of 3CY3-7m with TLG and TLoG, respectively. For TLoG, reactions occur on the timescale of seconds to a few minutes, with the consequence that no continuous trains of pulses were observed along with a 90% reduction in ZMW having a single molecule, in contrast to TLG (Figure 5A). Fluorescence traces start with pulses of low intensity in line with the fluorescence intensity of CY3 stacking to a C:8-oxoG base pair (Figure 2A), and change within a short period of time to shorter pulses at a higher intensity (Figure 6A), or terminate (Figure 6B). The nature of the products created is currently analysed by comparison of single molecule data with SPR and static fluorescence measurements using oligonucleotides modified with higher oxidation products of guanine including guanidinohydantoin (Gh), and spirodi(iminodihydantoin) (Sp), and 2-aminoimidazolone (Iz) [68,69,70]. When 5CY3-7m hybridised to TLG and TLoG, respectively, in both cases the dye stacks to a C:G base pair, and differences cancel out (Figure 7). A more detailed analysis of hybridisation of CY3-labelled oligonucleotides to TLG and TLoG will be presented in a forthcoming publication.

By removing the guanine donor from the C:G base pair, reactions were strongly reduced resulting in an increase in continuous trains of pulses from 11% (Bio34G) to 70% (Bio34ap) of the fluorescence traces upon hybridisation with 5CY3-7n measured over 25 min [20]. Interestingly, even in oligonucleotides lacking G, a considerable number of fluorescence traces with pulse pattern changes were found [71,72].

## 3. Discussion

The interactions of the dye with the terminal base pair are key for understanding the fluorescence properties of terminally CY3-labelled oligonucleotides in ds DNA. There is a dynamic equilibrium between unstacked and stacked conformations with prominent lateral motion in the plane of the base pair [33,34,73]. As shown by an NMR structure and molecular dynamics (MD) simulations, dye stacking leads to a close proximity of the electronic systems [34,35,74]. For 7 nt hybrids of TL derivatives, the interactions increase the affinity by approximately one order of magnitude compared to the unlabelled oligonucleotide [19]. This corresponds to a stabilisation by 7–8 kJ/mol for base pairs composed of a perfect match (C:G) and mismatch (C:A), as well as modified nucleobases (C:8-oxoG, C:I). The interactions are weak, in line with the nearly identical absorption spectra. According to our data from a large number of SPR measurements, hybrid stabilisation is a fundamental characteristic of dyes that do not carry two or more negative charges (Table 2, Table 3 and Table 4) [8]. The stabilisation energy determines the equilibrium position between open and stacked conformations and depends mainly on the dye charge and hydrophobic interactions. Stabilisation is largest for positively charged dyes and decreases with increasing negative charge. The loss of affinity through an increasing negative dye charge is mainly caused by a smaller association rate constant due to an increased repulsion by the negative charges of the probe backbone whereas the effect on hybrid dissociation is small, as demonstrated by means of the series of the symmetric cyanines (Supplement Table S1). The large stabilisation found for the hydrophobic neutral dye ATTO647N illustrates the effect of hydrophobic interactions.

Stabilisation is similar for CY3 interacting with a C:G (5CY3-7n) and a T:A base pair (CY3-7T) in the hybrid with Bio34G. CY3 attached to the 3′end of the oligonucleotide leads to a weaker stabilisation compared to the attachment at 5′, in agreement with the finding that CY3 is less efficiently stacking at 3′ [75]. The linker between oligonucleotide and dye also plays a role. In 5′CY3-7n, the dye is linked via a short aliphatic propyl chain (Figure 1) thus enforcing a close distance within stacked conformations [74]. The additional 18 atom polyethyleneglycol (PEG) linker in CY3-7nPEG increases the flexibility and allows the dye to adopt less constraint stacking conformations. The stabilisation found for 5CY3-7n/ Bio34ap indicates that in case of a missing opposite base (abasic site) the dye is stacking to a neighbouring base. This finding was supported by the observation of oxidation of G in the overhang by CY3 in single molecule measurements of Bio34G/ CY3-7T (see Supplement Figure S1 in [20]).

Dyes including DY-548, DY-549, and fluorescein having two or more negative charges have practically no affinity to the terminal base pair. SPR measurements were made at pH 7.4, at which fluorescein exists in an anionic and dianionic form (72%) [76]. A measurement at pH 5.9, at which mainly the monoanion exists (90%, pK_S_ ~6.9 for oligonucleotide conjugates) [76] leads to a small increase in the energy gain (1.7 kJ/mol) that is still smaller compared to dyes that have a single negative charge.

In all probe-analyte systems we investigated in which CY3 was stacking to a C:G base pair, including TLG, Bio34G and derivatives, and in 15 nt probes containing a single G, single molecule fluorescence trace analysis revealed the formation of 8-oxoG, among other (unidentified) products [20]. The mechanism of guanine oxidation was well investigated by a number of groups for its role in DNA damage in cells by reactive radical species and high energy irradiation [52,77,78,79,80,81,82,83]. The reaction was found to proceed by oxidation of G to G^+^, followed by the addition of water or oxygen [84,85,86]. It is reasonable to assume that, in a close donor–acceptor pair, G^+^ is created by PET to the dye, therefore the detection of 8-oxoG is a strong indication of fluorescence quenching. Furthermore, the probe–analyte systems under study showed the typical characteristics of electron transfer including the dependence on the oxidation potential of the donor, as demonstrated by using the example of an abasic site, G, G trimer, and 8-oxoG, respectively, and a distance dependence in cases where the dye was stacking to a neighbouring T:A base pair, as in Bio34G/CY3-7T. Measurements conducted in the presence of DTT excluded a photooxidation type II [20,87].

Electronic coupling as a precondition of electron transfer is ensured by the close proximity of G donor and CY3 acceptor due to stacking interactions. Experimental results and calculations demonstrated a large gain of G oxidation potential in particular upon base pairing due to formation of the neutral G radical, G(−H)^∙^:C(+H)^+^, by intra-base pair proton transfer. A stabilisation in the order of 0.58 to 0.75 eV as calculated by Colson and Hutter is expected to result in a sufficiently large negative free energy of electron transfer [48,49,88], that compensates for the relatively high negative reduction potential of CY3 [39,89]. This is in line with findings from single molecule measurements determining the dependence of product formation on the oxidation potential of the stacking base pair (see above). A quantitation is not possible because, to the best of our knowledge, redox potentials of oligonucleotide hybrids and dye conjugates are not available.

The fluorescence intensity of TL derivatives hybridised with CY-26 in Figure 2A follows the trend of decreasing oxidation potentials of the stacking base pair in the order I < A < G < 8-oxoG [29,61,66], in line with the idea of fluorescence quenching via PET. Differences in intensity between G and 8-oxoG vanished when the dye was not directly stacking to C:X as demonstrated for CY3-27/TLX hybrids (Figure 2B). The additional T:A base pair increases the distance between C:X and dye, thus decreasing the extent of electronic coupling [90]. As a consequence, the dye is “blind” for chemical reactions occurring at distant sites within a hybrid, for example after an intramolecular charge transfer from a distant GG donor as in the sequence of Bio34G. This explains the observation that in single molecule measurements a large number of fluorescence traces were found to feature transitions from long to short pulses without a change of intensity (see for example Figure 3b–d in [20]). The larger intensity found for TLA, we attribute to a steric effect caused by the disturbance of the double strand conformation by the C:A mismatch, which affects dye stacking [59].

Based on these findings, we conclude that fluorescence of CY3-labelled oligonucleotides is governed by an interplay of two opposite effects. Firstly, the steric effect of blocking trans-cis isomerisation upon interaction with a nucleobase or base pair, respectively, that causes an increase in fluorescence efficiency. Secondly, the electronic effect of fluorescence quenching as a result of the overlap of the electronic systems of base pair donor and dye acceptor. In dye-labelled nucleosides and ss oligonucleotides, the first effect prevails because of the weak donor properties of the nucleobases [3,29]. In contrast, in ds DNA the nucleobase pairs are much better electron donors, leading to stronger fluorescence quenching upon hybridisation. A precondition for this is an efficient stacking in light of the poor acceptor properties of CY3. Moreover, the presence of negative charges in the sulfonated derivatives is expected to decrease the reduction potential and, correspondingly, the free energy of electron transfer [91]. This explains why in Stern–Volmer quenching experiments of (disulfonated) CY3 with nucleoside monophosphates, an increase in fluorescence intensity was observed [39]. In the CY3-26/TLX hybrids investigated, the quenching effect is predominant over the reduced isomerisation efficiency and leads to the observed intensity decrease. The combined effect of a reduced stacking energy and a decreased ΔG_ET_ was observed in single-molecule hybridisation experiments of Bio34G with the disulfonated derivative DY547-7n by a strong reduction in product formation compared to 5CY3-7n [19]. Reports on an increased fluorescence intensity upon hybridisation indicate that in this particular system, the dye is not efficiently stacking to the terminal base pair [16].

The concept of donor–acceptor interactions can be used to explain the low fluorescence quantum yield observed for ss CY3–5′poly(dA) and poly(dAdG) by Harvey [4]. Using MD simulations, dA_19_ was shown to adopt elongated conformations with nearly fully stacked and strongly interacting bases [92]. In ss dA and also dG oligomers, stacking was demonstrated experimentally to decrease the oxidation potential as a result of charge delocalisation [93,94,95]. As for the model systems investigated in this study, this leads to an increase in fluorescence quenching. This example illustrates the general validity of the notion of fluorescence quenching to explain the fluorescence properties of CY3 and other dyes when interacting with DNA [32,96]. Fluorescence quenching upon hybridisation was also reported in rhodamine and fluorescein labelled oligonucleotides, and in complexes of CY3-labelled oligonucleotides with proteins [32,97,98,99].

## 4. Materials and Methods

All measurements were performed in HBS buffer (150 mM sodium chloride, 10 mM HEPES, 3 mM EDTA, 0.05% *v*/*v* Tween-20, pH 7.4, Teknova, Holister, OH, USA) at 20 °C. Ultrapure and filtered water (“MilliQ”, Millipore, Billerica, MA, USA) was used for the preparation of all solutions. HPLC purified oligonucleotides were from Microsynth and used as received (Balgach, Switzerland). ATTO532 was from Eurofins Genomics. Dye conjugates with 7mers were labelled by the company by coupling the dye N-hydroxysuccinimide (NHS) ester to the hexylamine modified oligonucleotide. The ester groups were attached to the dye via short alkyl chains (C_1_: CY3B, C_3_: Methylene Blue, DY-530, DY-547, DY-548, DY-549, ATTO532, ATTO550, ATTO647N, C_5_: CY3, CY5, DY630, directly coupled to amid/sulfonamid: FAM, TAMRA, Texas Red; for molecular structures and linker see https://www.microsynth.ch/dyes.html). For labelling with 3′CY3, a CY3 modified solid support was used (www.linktech.co.uk/products/labelling/cyanine_dyes/1320_3-cyanine-3-synbase-cpg-1000-110). The structure of the DY-547 oligonucleotide conjugate is given in Figure 3 in [19]. The sequence of TL was taken from the literature [100]. TL derivatives (TLX) were modified at a single position by substituting a G with 8-oxoG, A, and I, respectively. CY3-7nPEG additionally contains an 18 atom hexaethyleneglycol linker between oligonucleotide and dye. Sequence data are given in Table 5.

### 4.1. Optical Measurements

Absorbance spectra of 10 µM solutions of CY3-26 hybridised with TL derivatives were measured using a DS-11 instrument (Denovix, Bucher, Basel, Switzerland). Fluorescence spectra were recorded using a Safire2 plate reader (Tecan, Männedorf, Switzerland) with sample volumes of 100 µL in black Greiner 96-well plates. Hybrids of 200 nM CY3-26 with 1 µM TL derivatives were excited at 510 nm with monochromator slits set to 10 nm/10 nm for excitation and emission, respectively, between 530 and 750 nm at a spectral resolution of 1 nm. For sample preparation, solutions were heated for 2 min at 80 °C and allowed to cool slowly in the thermomixer. Non-matching ON34 was used as a negative control. Mean intensities and the error calculated from three measurements were plotted.

### 4.2. Surface Plasmon Resonance Measurements

A Biacore T200 instrument (Cytiva, Freiburg, Germany) was used for SPR measurements. The experimental setup was described in a previous publication [19]. In a first step, chip surfaces were coated with neutravidin following the recommendations of the manufacturer. TL derivatives (TLX) were hybridised to a biotinylated 25 nt oligonucleotide containing three locked nucleic acid (LNA)-modified nucleotides in order to increase hybrid stability. Bio25LNA/TLX hybrids (X=G, 8-oxoG, A, I) were prepared by heating a 4 µM solution of Bio25LNA with 6 µM of TLX in HBS buffer for 2 min at 80 °C and cooled slowly. Hybrids were immobilised to a 2D carboxylated tetraethylene glycol chip surface (CMTEG, Xantec, Düsseldorf, Germany). Probe surface densities were 350–400 RU. TLI was measured at 15 °C using a 3D carboxymethyldextrane surface (CMD200M, Xantec) at a probe density of 1700 RU in order to gain sufficient signal. The biotinylated Bio34 was immobilised to a CMD200M surface at probe densities of 110 RU. Bio34r was measured on a 2D carboxymethyldextrane surface (CMDP, Xantec) at a probe density of 210 RU. For kinetic measurements, 8 dilutions of the analytes were injected in duplicate, and for referencing, there were 4 additional injections of HBS buffer. A neutravidin-coated flow cell was used as reference channel. Equilibrium constants *K*_D_ were determined from equilibrium signal intensities because they are not affected by the rebinding effect [19]. For data evaluation, BiaEvaluation software (v.2.0.3) was used.

### 4.3. Single-Molecule Measurements

A modified single-molecule RSII+ sequencer (Pacific Biosciences, Menlo Park, CA, USA.) was used for kinetic measurements. Experimental setup and data evaluation were described in a previous publication [19]. TLG and TLoG, respectively, were hybridised to a biotinylated 25 nt oligonucleotide containing three LNA modified nucleotides, and immobilised at the SMRT chip surface at 125 pM in HBS for 90 s. Bio34 and derivatives were directly immobilised at the surface at 30–60 pM for 90–150 s. Fluorescence traces of hybridisation of 20–100 nM CY3-labelled analytes in HBS were monitored over a period of 15–25 min.

## 5. Conclusions

The concept of fluorescence quenching in oligonucleotide dye conjugates is useful to explain the fluorescence properties of CY3 stacking to a terminal base pair. The quenching arises from the fact that donor properties of nucleobases are subject to a dramatic change upon base pairing and stacking, and the close proximity of the donor and acceptor due to formation of a weak complex. Single-molecule experiments demonstrated that CY3 is a potent oxidant to guanine. For this reason, care must be taken with the design of oligonucleotide conjugates to ensure that, in a hybrid, the dye is not placed in the vicinity of guanine, especially not stacking to a C:G and G:C base pair, respectively. In order to reduce stacking interactions, sulfonated derivatives such as DY-547 should preferably be used.

It can be concluded that, firstly, the redox properties of single nucleobases in ds DNA must be seen in the context of the whole entity of the base pair within the stack of bases, and the influence of adjacent base pairs. Secondly, stacked nucleobase pairs in ds DNA are much better electron donors (and acceptors) than nucleobases in ss oligonucleotides. And thirdly, the use of redox potentials of isolated nucleobases is an inappropriate approximation, only suitable for characterising relations between bases. Redox properties are essential for understanding the behaviour of CY3 and other dye conjugates interacting with DNA; the lack of fundamental electrochemical data is unsatisfying, however, which prompted us to tackle the experimental determination of redox potentials of dye-labelled oligonucleotides and hybrids, as well as measurements of time-resolved fluorescence.

## Figures and Tables

**Table 1:** Absorption and fluorescence maxima of CY3-26 and hybrids with TL derivatives and ON34.

Sample	λ_max_ (abs)	λ_max_ (fl)
CY3-26	550	566
TLG	549	564
TLoG	549	565
TLA	550	565
TLI	549	566
ON34	550	565

**Table 2:** Equilibrium constants *K*_D_ for TL derivatives hybridised with CY3-7m and CY3-8mG, and unlabelled compounds, 7m and 8mG, respectively, on a CMTEG surface in HBS buffer at 20 °C. *K*_D_ ratios and free energy gain ∆G resulting from dye labelling was calculated using the van’t Hoff equation. TLI was measured on a CMD200M surface at 15 °C.

Probe	Analyte	*K*_D_/nM	*K*_D_ Ratio	∆G/kJ/mol
TLG	7m	2230(90)		
	3CY3-7m	107(3)	20.8	7.4
	5CY3-7m	77.4(23)	28.8	8.2
TLoG	7m	4401(229)		
	3CY3-7m	253(6)	17.4	7.0
	5CY3-7m	155(3)	28.4	8.2
TLA	7m	9475(1832)	27.3	8.0
	3CY3-7m	347(12)		
TLI	7m	8832(866)	28.8	8.0
	3CY3-7m	307(5)		
TLG	8mG	320(16)	12.2	6.1
	3CY3-8mG	26.2(20)		
TLoG	8mG	230(13)	10.6	5.8
	3CY3-8mG	21.6(24)		

**Table 3:** Equilibrium constants *K*_D_ for Bio34 derivatives hybridised with various dye-labelled 7nt oligonucleotides on a CMD200M surface in HBS buffer at 20 °C, the *K*_D_ ratio (unlabelled/labelled analyte), and free energy gain ∆G resulting from dye labelling, calculated using the van’t Hoff equation. Bio34r was measured on a CMDP surface. MB: methylene blue; FAM: fluorescein.

Probe	Analyte	Dye Class	Dye Charge	*K*_D_/nM	*K*_D_ Ratio	∆G/kJ/mol
Bio34G	7n	-	-	978(25)	-	-
	5CY3-7n	sym. cyanine	+1	32.8(41)	29.8	8.3
	3CY3-7n	sym. cyanine	+1	46.4(15)	21.1	7.5
	CY3-7nPEG	sym. cyanine	+1	84.6(56)	11.6	6.0
	CY3B-7n	sym. cyanine	0	92.9(42)	10.5	5.8
	DY547-7n	sym. cyanine	−1	206(6)	4.5	3.7
	DY530-7n	rhodamine	−1	217(9)	4.5	3.7
	TAMRA-7n	rhodamine	0	278(5)	3.5	3.1
	TexasRed-7n	rhodamine	0	94.0(51)	10.4	5.7
	ATTO532-7n	rhodamine	−1	200(8)	4.9	3.9
	ATTO550-7n	rhodamine	+1	39.1(44)	25.0	7.9
	CY5-7n	sym. cyanine	+1	28.8(17)	34.0	8.6
	DY630-7n	asym. cyanine	0	84.4(27)	11.6	6.0
	ATTO647N-7n	carbopyronine	0	32.2(41)	30.3	8.4
	MB-7n	phenothiazine	+1	29.6(18)	33.0	8.6
	FAM-7n	fluorescein	−2	881(69)	1.1	0.3
Bio34r	7r	-	-	506(210)	-	-
	DY548-7r	sym. cyanine	−2	423(49)	1.2	0.4
	DY549-7r	sym. cyanine	−3	534(68)	0.95	−0.1

**Table 4:** Equilibrium constants *K*_D_ for Bio34 derivatives hybridised with 7n, 7T and 5CY3-7n, 3CY3-7n, and CY3-7T, respectively, on a CMD200M surface in HBS buffer at 20 °C. *K*_D_ ratios and free energy gain ΔG resulting from dye labelling was calculated using van’t Hoff equation.

Probe	Analyte	*K*_D_/nM	*K*_D_ Ratio	∆G/kJ/mol
Bio34G	7T	3025(68)	19.7	7.3
	CY3-7T	153(3)		
Bio34I	7n	7419(61)	28.8	8.2
	CY3-7n	257(10)		
Bio34U	7n	14,830(494)	7.4	4.9
	CY3-7n	1987(42)		
Bio34ap	7n	45,111(24,127)		
	CY3-7n	15,015(6605)	3.0	2.7
	3CY3-7n	1744(124)	25.9	8.0

**Table 5:** Sequences of oligonucleotides used. The binding sequences of 7m, 7n, and 7r, respectively, are underlined. Sequence modifications in TL and Bio34 derivatives are marked in bold. Bio25LNA contains LNA at the positions marked in bold. Hybridised to the TL derivatives, it was used for immobilisation to the surface plasmon resonance (SPR) chip surface, the hybrid region is marked with a dashed line. CY3-26 and CY3-27 hybridise to the TL derivatives forcing the dye to interact with the base marked in bold. ap: abasic site; G°: 8-oxoG; DYE: CY3B, DY-547, DY-530, DY-630, CY5, TAMRA, Texas Red, ATTO532, ATTO550, ATTO647N, methylene blue, fluorescein. Bio34G and 7n are identical with Bio34 and 7mer, respectively, used in previous publications [19,20].

Oligonucleotide	Sequence
TLG	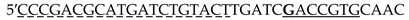
TLoG	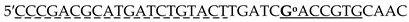
TLA	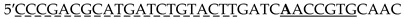
TLI	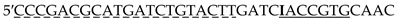
7m	5′CACGGTC
5CY3-7m	5′CY3–CACGGTC
3CY3-7m	5′CACGGTC–CY3
8mG	5′GGCACGGTG
3CY3-8mG	5′GCACGGTG–CY3
Bio25LNA	5′AGTACAGATCATGCGTCGGGTTTTT-Biotin
CY3-26	5′CY3–CGATCAAGTACAGATCATGCGTCGGG
CY3-27	5′CY3–TCGATCAAGTACAGATCATGCGTCGGG
ON34	TTTTTGGAAACTGTATTGGCACTGAGTAGACTCC
Bio34G	5′Biotin-TTTTTGGAAACTGTATTGGCACTGAGTAGACTCC
Bio34I	5′Biotin-TTTTTGGAAACTGTATTGGCACTIAGTAGACTCC
Bio34U	5′Biotin-TTTTTGGAAACTGTATTGGCACTUAGTAGACTCC
Bio34ap	5′Biotin-TTTTTGGAAACTGTATTGGCACTapAGTAGACTCC
7n	CAGTGCC
5CY3-7n	5′CY3-CAGTGCC
3CY3-7n	5′CAGTGCC-CY3
DYE-7n	5′DYE-CAGTGCC
7T	5′CY3-TCAGTGC
CY3-7T	5′CY3-TCAGTGC
Bio34r	5′Biotin-TTTTTGGAAACTGTATTGTCACGGAGTAGACTTT
7r	5′CCGTGAC
DY548-7r	5′DY548-CCGTGAC
DY549-7r	5′DY549-CCGTGAC

## Supplementary Materials

The following are available online, Table S1: Kinetic data of hybridisation of dye-labelled analytes, measured in HBS at 20 °C.
