# Supplementary material for: Dependence of Fluorescence Quenching of CY3 Oligonucleotide Conjugates on the Oxidation Potential of the Stacking Base Pair
Source: Molecules. 2020 Nov 17;25(22):5369. doi: 10.3390/molecules25225369 (PMC7698394; doi:10.3390/molecules25225369)
Supplement: Supplementary file 1 [file molecules-25-05369-s001.pdf]

Supplementary Materials: The following are available online at www.mdpi.com/xxx/s1, Table S1: Kinetic data of hybridisation of dye-labelled analytes, measured on a CMD200M surface in HBS at 20°C.

Table S1: Kinetic data of hybridisation of dye-labelled analytes measured on a CMD200M surface in HBS at 20°C.

**Probe**	**Analyte**	k_{on}**/ 10⁵ mol⁻¹s⁻¹**	k_{off}**/ s⁻¹**	K_D**/ nM**	**RU(max)**	χ^2
Bio34	CY3-7n	18.42(10)	0.0253(2)	13.8	4.6	0.03
	3CY3-7n	11.58(4)	0.0410(1)	35.0	20.3	0.07
	CY3-7PEG	10.61(7)	0.0566(2)	53.3	4.6	0.03
	CY3B-7	8.53(3)	0.0495(1)	58.0	10.3	0.02
	DY547-7	3.17(1)	0.0592(1)	186	21.2	0.04
	DY530-7	2.74(1)	0.0346(1)	126	7.3	0.02
	TAMRA-7	5.47(7)	0.1006(10)	184	3.5	0.03
	TexasRed-7	13.45(5)	0.0582(2)	43.2	4.8	0.08
	ATTO532-7	9.88(3)	0.142(1)	200	10.6	0.01
	ATTO550-7	18.52(4)	0.0337(1)	18.2	10.5	0.02
	CY5-7	38.1(3)	0.0819(6)	21.5	9.1	0.04
	DY630-7	9.91(5)	0.0561(2)	56.5	6.7	0.04
	ATTO647-7N	37.1(1)	0.0589(1)	15.9	7.0	0.03
	MB-7	9.36(3)	0.0187(1)	20.0	22.1	0.20
	FAM-7	4.02(2)	0.1653(1)	412	11.5	0.02
Bio34r	DY548-7	1.45(1)	0.0435(2)	301	5.3	0.03
	DY549-7	1.44(2)	0.0459(2)	319	3.6	0.03
TLG	3CY3-7m	13.81(14)	0.1564(1)	113	10.3	0.10
	5CY3-7m	14.74(13)	0.0847(1)	57.5	9.5	0.05
TLoG	3CY3-7m	17.03(13)	0.2758(2)	162	5.0	0.01
	5CY3-7m	13.28(10)	0.1387(1)	104	8.7	0.06
